# Multi-Specialty Care for Second-Degree Pressure Cooker Explosion Burn Injuries

**DOI:** 10.51894/001c.17738

**Published:** 2020-10-30

**Authors:** Casey Schukow, Billy R. Nordyke

**Affiliations:** 1 Michigan State University College of Osteopathic Medicine; 2 Nodryke Family Medicine Henry Ford Health System

**Keywords:** multispecialty care, burn scarring, partial and full-thickness burn wounds, pressure cooker explosion

## Abstract

**CONTEXT:**

Although pressure cookers are very common kitchen utensils used in the United States, only a few cases of serious injuries secondary to pressure cooker explosions have been reported in the medical literature. When second-degree (i.e., “partial-thickness”) burns result from pressure cooker explosions, wounds involving near to or greater than 10% of total body surface area typically require multidisciplinary treatment, with burn center referral for proper wound care, potential fluid resuscitation, and eventual scar management.

**EXAMPLE CASE:**

The example patient described in this report was an African American female in her early 30s who presented during the summer of 2020 after suffering varying levels of second-degree burns to her bilateral upper torso and left wrist (i.e., approximately 10%, total body surface area). The authors first saw the patient during a primary care office visit a week after her initial injury when she first went to a local urgent care clinic. Upon her arrival to the second author’s family medicine clinic, a multi-specialty wound recovery plan was initiated since her first urgent care visit treatment had been minimal without prophylactic antibiotic therapy or placement of a burn center referral.

**CONCLUSIONS:**

Partial and full-thickness burn injuries generally warrant immediate clinical (i.e., body surface area burn assessment, fluid resuscitation, empiric antibiotics) as well as ongoing (burn center referral, debridement procedures, active scar management, provision of psychological support) treatment needs. This paper discusses the critical opportunities posed for more extensive burn patients’ physicians to first categorize the extent of burn wounds and initiate subsequent specialty care in other settings.

## INTRODUCTION

Found in approximately one-fifth of households, pressure cookers are one of the most commonly used cooking devices in the United States.^1^ These airtight utensils intended for quick cooking operate by bringing boiling water from 100^O^ C. to roughly 121^O^ C. at a standard pressure of about 15 pounds per square inch.^1^ As pressure builds, the cooker lid is designed to remain secured through both locking and safety valve mechanisms.^1^ Although the medical literature indexed on PubMed regarding severe burn injuries secondary to pressure cooker explosions has remained minimal, the complexity of burn care coordination management has appeared in multiple articles.^1–5^

Burn wounds are generally classified by their extent of skin layer involvement since this information is important to understand to order varying degrees of intervention. Superficial, or “first-degree,” burns are commonly associated with sun-bathing or salon-tanning and involve only the top epidermis of the skin.^6,7^ Although these types of lesions are often both erythematous (i.e., reddened) and painful, they typically heal well with the use of topical anti-inflammatory agents such as aloe vera agents that promote skin-regeneration without scarring.^7,8^

Partial thickness, or “second-degree”, burns involve the epidermis as well as the dermis and are classically characterized by blister formation with possible blood vessel exposure.^6,7^ The dermis can be further subcategorized into a superficial, or “papillary”, level, and a deep, or “reticular”, level. The deeper a burn penetrates the dermis (i.e. superficial dermis versus deep dermis), the less likely it will blanch with pressure. Even though dermal nerve fibers often remain intact, most second-degree burns induce extreme pain.^6,7^

Full thickness, or “third-degree”, burns involve all layers of the skin and may involve underlying subcutaneous muscle and bone.^6,7^ These burns often overtly appear on a spectrum from white to black and are likely to not be as painful as they may have destroyed dermal nerve fibers.^7,9^ Since both second and third degree burns destroy the epidermal regenerative cells of the stratum basale, both injuries will be replaced with scar tissue.^6,7,10^

As mortality rates secondary to severe burn wounds have decreased over time, a major component of burn wound care is treating burn scars which can lead to mental, physical, and social distress.^11^ Scars may contract leading to impaired mobility of the affected region, and hypertrophic and keloid (i.e., fibrous tissue) scars result from inappropriate collagen tissue outgrowth in response to the wound healing process.^12^

Hypertrophic scars can be effectively treated with long acting injected corticosteroids such as triamcinolone acetonide.^11,12^ Corticosteroids are believed to decrease excessive collagen synthesis through attenuating fibroblast and keratinocyte hyperactivity.^11,12^ However, well-documented side effects such as dermal atrophy and skin hypopigmentation, especially in African American patients, make corticosteroid treatment questionable from a cosmetic perspective.^11^

Other potential therapies for the management of burn wound scarring include silicone gel, transforming growth factor-β modulators, fat grafting, and laser therapy.^13^ Although it may not prevent scar formation, intralesional injections of the neurotoxin Botulinum toxin A serve to suppress acetylcholine release in surrounding muscle and, thus, decrease wound contraction.^13^

Recent studies have indicated that polymers such as elastin and silk may improve the tissue regeneration seen in burn wounds by promoting a proper scaffold for keratinocyte maintenance, increasing appropriate fibroblast-mediated collagen synthesis, decreasing myofibroblast contractile activity, and up-regulating angiogenesis (i.e., the generation of new blood vessels) within the scar tissue.^13^

With impeded psychological distress a primary concern in nearly three-quarters of adolescents and adults with scarring burn wounds, especially in cosmetic areas such as the face and upper chest, understanding scar formation and treatment are essential elements of burn wound management.^13^ It is of similar importance for clinicians to acknowledge the likelihood of scar formation to patients and provide an empathetic environment for them as they receive their prognosis. Pertaining to osteopathy, it is essential to remember to console patients and treat their wholistic needs, just as much as other underlying diseases or conditions.^14^

Less extensive second-degree burns can often be managed in clinic settings with a combination therapy of exposure to cool water, daily dressings with antibiotic ointments, or creams and pain management. In contrast, the management of third-degree burns is typically more intense.^9^ In 2020, the American Burn Association recommended that burn patients with any extent of third-degree burns or wide-spread second-degree burns be referred to specialize burn centers for proper wound therapy such as debridement (i.e., removal of dead, damaged, or infected tissues) procedures and empiric antibiotic treatments.^9,15^

As most burns are often occur at a mix of different depths, both eliciting a thorough injury history during the patient’s initial presentation with close lesion inspection are essential for the proper care and management of burn wound victims.^16^

## EXAMPLE PATIENT DESCRIPTION

**Figure 1.**
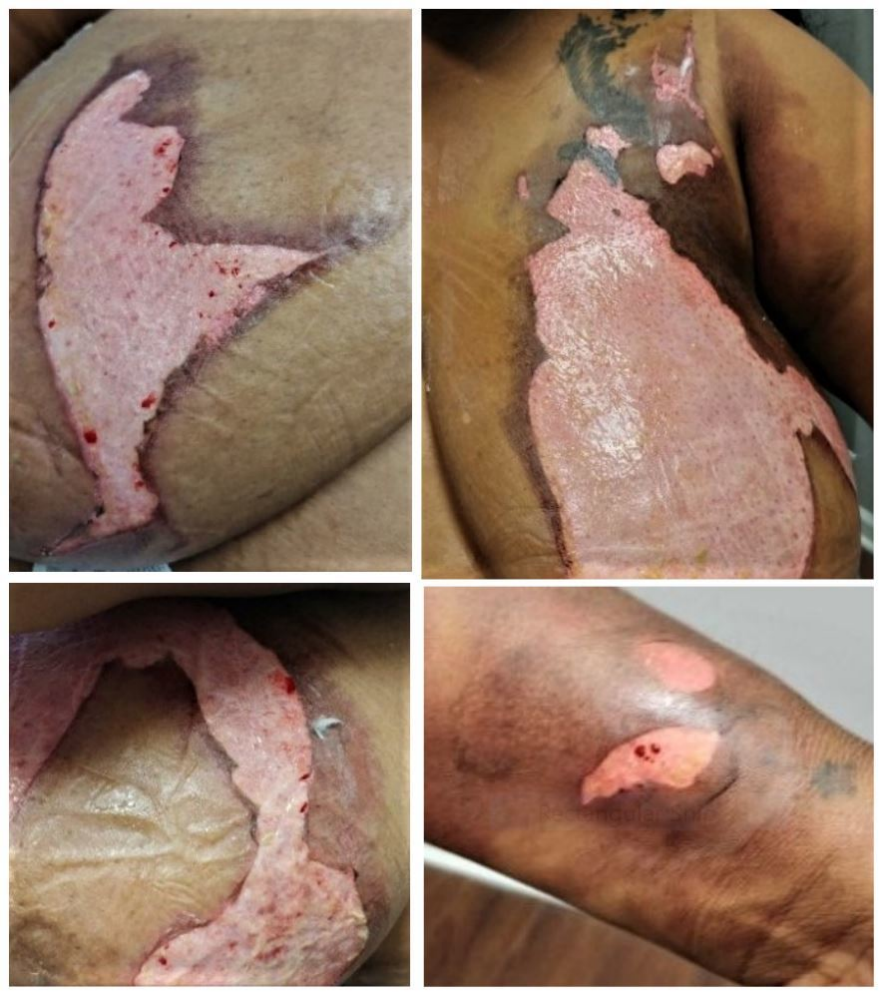
Photographic images of patient’s second-degree burns on her upper torso (upper Right, Lower Left) and her wrist (lower Right). These photographs were taken after the patient signed a written consent form, reviewed the final cropped photos and approved each for SMRJ publication per written consent.

During the summer of 2020, an African American female in her early thirties presented to us at the second author’s community-based clinic with extensive burns covering her upper torso and left wrist. (Figure 1) A week prior, the patient had used a pressure cooker when its top exploded in front of her chest and tipped over, releasing steam and boiling water onto her skin. Immediately after suffering her burns, the patient admitted to falling into a state of severe distress and reported having been escorted into a cold shower by her significant other to soothe her burns.

Later that day, this patient went to a local urgent care clinic where she was diagnosed with second-degree burns of the bilateral upper torso and left lateral wrist (Figure 1). At that first visit, she was administered an intramuscular Ketorolac (Toradol) injection to treat her pain. She was sent home with oral Diazepam (i.e., Valium) tablets to treat her anxiety and keep her calm. However, she reported that she did not receive antibiotic prophylaxis at the visit, nor was she discharged with wound care dressing or instructions. Although authors have recently explained that not every patient with second-degree burns requires prophylactic antibiotic therapy, the authors concluded otherwise given the extent of this woman’s injured body surface area.^17^

At the first visit to the authors’ clinic a week later, she denied having any fever, chills, fatigue, or dizziness since her initial urgent care visit. She reported having self-managed her wounds with daily re-coverings of non-adhesive dressing and tape. Her comorbidities were unremarkable, except for a “severely overweight” body mass index (BMI) of 39.6 kg/m^2^.

After removal of her dressing in the second author’s clinic, silver sulfadiazine 1% topical cream (i.e., Silvadene) was applied over the burn lesions, which covered approximately 10% of her total body surface area and accompanied with areas of pinpoint bleeding. Kerlix antimicrobial bandage gauze coverage was initiated. Although newer occlusive dressings may result in faster healing rates requiring fewer dressing changes, silver sulfadiazine remains a standard topical antimicrobial treatment for second-degree burns.^18^

The patient was released with prescriptions for a 400 mg. jar of the silver sulfadiazine 1% cream to be applied once daily (i.e., cover 1/16’’, full-thickness), 150 Kerlix bandage units, as well as a prescription for 500 mg capsules of Cephalexin (Keflex) for her to take every six hours for seven days for wound infection prophylaxis. The prescription was sent to her nearby pharmacy and she confirmed during a later follow-up phone conversation that she had taken this medication as prescribed.

She was discharged with instructions to follow-up with a burn center for further care and management of her wounds. During the initial office visit, we provided her with specific contact information for a nearby burn center that she confirmed she had the means to get to. This referral decision was guided by 2010 recommendation of the American Burn Association, indicating that patients with partial thickness burns greater than 10% total body surface area (TBSA) and pre-existing medical conditions (e g., higher BMI) that could complicate management and recovery should be referred to a burn center.^15^

In order to manage the patient’s pain, she was also written a three-day prescription of hydrocodone 10 mg-acetaminophen 325 mg (i.e., Norco, Vicodin) tablets instructed for one pill to be taken up to three times a day orally. This pain medication was prescribed for her after we had reviewed her record on the Michigan Automated Prescription System (MAPS) for documented patient drug-seeking behavior.^19^ We reviewed with the patient how to properly resume home care management of her wounds and medications, as well as scheduling her a referral for follow-up care or immediate discharge to a local emergency department if her wounds worsened.

The worsening symptoms in this patient suggesting possible bacterial invasion included, but were not limited to, fever, chills, tachycardia, hypotension, palpitations, and increased erythema and tenderness surrounding her wounds. Upon further questioning, the patient wondered about the extent of her lesions, as well as subsequent complications and possibility of scarring. We conveyed similar concerns and try to validate her worries, eliciting patient agreement that she would be diligent in adhering to our discharge instructions and burn center referral.

Since the initial visit, we had followed up with her several times over the phone to ask her about her status and wound care. The patient confirmed that she was continuing to use Silvadene and wet-dry gauze application cycles over the phone, although we did not receive records of her following-up with the wound care clinic until she visited the second author at an in-person appointment nearly two months after the initial incident.

**Figure 2.**
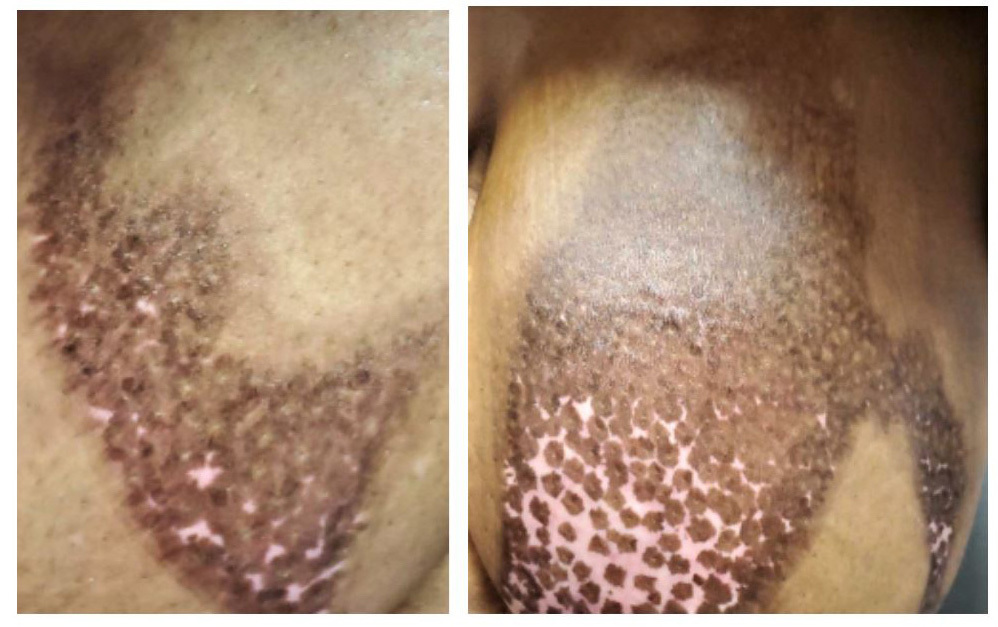
Two-month follow-up of Patient’s Burn Injuries. Photographs obtained after patient signed written consent, reviewed cropped photos and agreed to SMRJ publication per written consent.

During this visit, she stated that the professionals at the wound care clinic did not believe she needed any grafting to aid in the repair of burn wounds. Instead, she said they would continue to debride her wounds of any necrotic tissue, while re-applying alginate-based dressings coupled with the Silvadene cream that she was initially prescribed. After nearly two months of therapy, she admitted that her wounds were healing “nicely.” She has been generally happy with the treatments and all the supports that she had received. (Figure 2)

Alginate is a non-toxic, biocompatible polymer that is naturally obtained from brown seaweed.^20^ Alginate dressings are used often in burn wound care as they absorb excess fluid, maintain a moist environment, and decrease the occurrence of bacterial infections at wound sites.^20^

Although we would have liked to have followed-up with the patient in the clinic sooner than two months, we are overall pleased with the progress she has made. The phone conversations we had with her aided in gauging this woman’s condition (i.e., if she felt like her conditioning was getting worse, she had our support to come back to the clinic sooner for needed guidance). Upon finally seeing her and her wounds in person, we again offered her words of affirmation and encouragement, acknowledging that she was positively responding to the Silvadene and dressing therapy.

## DISCUSSION

In this patient’s case, various portions of her lesions were accompanied with pinpoint bleeding as well as diffuse pain, thus leading to our diagnosis of an extensive second-degree burn rather than a first or third-degree injury. When caring for burn victims in such clinic-based settings, it is essential to get an approximate TBSA prediction, as partial thickness burns greater than 20% TBSA in adults generally warrant burn care center referrals to help reduce healing complications.^15^

In adults with second or third-degree burns greater than 20% TBSA, it has been shown that failure to initiate immediate fluid resuscitation can lead to myoglobinuria (i.e., muscle cells in urine as a results of injury to tissue), hemoglobinuria (i.e., hemoglobin in urine), multi-organ failure (i.e., especially renal), and, ultimately, mortality.^16^ Partial and full-thickness burn wounds also degrade the Type 1 collagen located within the sub-epidermal levels of the skin, requiring adequate protein replacement.^16^ Although the extent of this patients’ wounds were less than 20% TBSA, we had still concluded that a burn center referral was appropriate given the location of her wounds and higher likelihood of complicated healing secondary to her high BMI.

**Figure 3.**
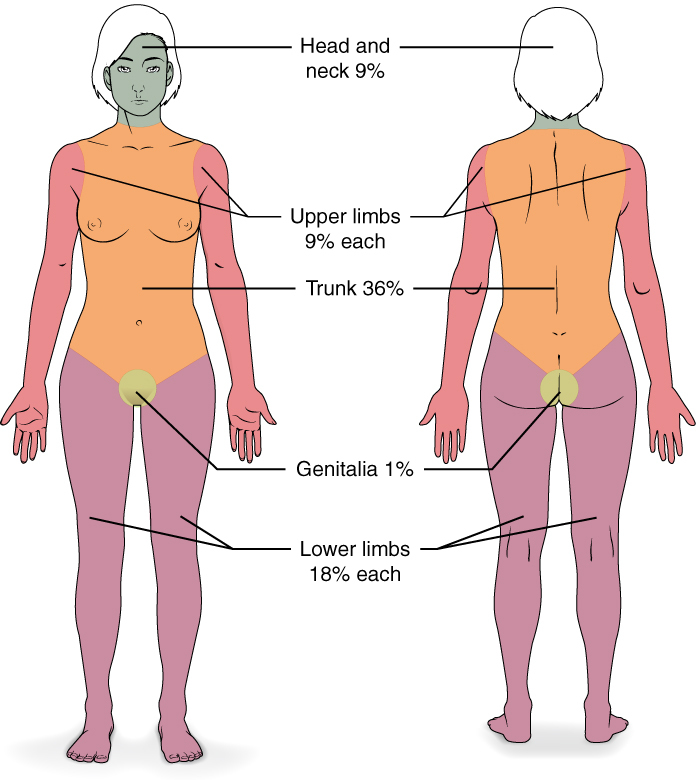
Diagram of the Wallace Rule of 9s. Retrieved via public domain Wikipedia.^22^

The “Wallace Rule of 9s” provides a quick estimate for physicians if presented with burn wound patients, to foster guidance on whether or not referral to a burn unit is indicated (Figure 3).^16^ The palm is the mainstay of TBSA prediction, which represents approximately 0.5% TBSA (i.e., without including the fingers or thumb).^21^ Using the groin as an example observing this method, a patient’s groin would be estimated to be about 1% TBSA, or equivalent to two of his or her palms.^16,22^

Again, the “Wallace Rule of 9s” is meant to provide a rough estimate of TBSA for adults of ideal height with BMI ranges of between 18.5 to 24.9 BMI.^16^ Obese patients (>30 BMI), however, begin to carry excess weight and surface areas disproportionately.^16^ One 2013 study demonstrated how the surface areas of the torso, arms, and legs in obese patients are closer to 52%, 7%, and 15%, respectively; as opposed to Wallace’s estimates of 36%, 9%, and 18%.^23^ Observing this method, we had approximated a 10% TBSA in this patient by estimating 7% burn-surface area on the anterior portions of her left upper torso, approximately 3% surface area on the right upper torso and about 0.5% on her left wrist (Figure 1).

**Figure 4.**
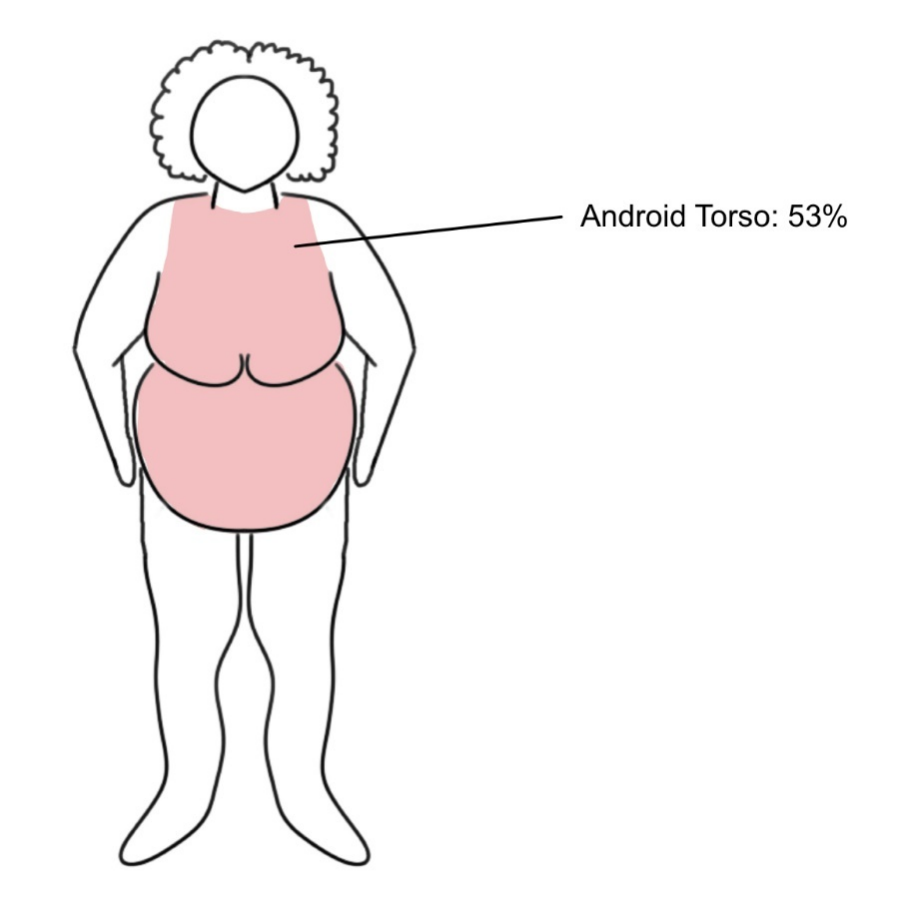
Diagram of “Android” body shape with approximate torso body surface area designation as estimated by the 2013 study referenced above. Drawn using Sketchbook app of iPad Pro, courtesy of Lauren Noel Cole.

This same 2013 research group proposed classifying obese patients into upper-body and lower-body predominate excess body mass categories.^23^ The former were referred to as “Android”-shaped and had an average torso surface area of 53% (Figure 4), while the latter were referred to as “Gynecoid”-shaped and had an average torso surface area of 48%.^23^

This assessment of varying distributions of TBSA brought on by Williams and Wohlgemuth was essential in how we cared for our patient as she had a BMI of nearly 40 with an “Android” body shape.

In other words, the upper chest burns on this “severely overweight” patient (i.e., 39.6 BMI, TBSA approx. 10%) warranted enough concern to be needed for burn unit referral. Fortunately, this woman had not sustained co-existing injuries to her orbit, mandible, or upper airway, all of which would have required immediate referral to a traumatic burn unit or emergency department depending on the severity of presenting symptoms.^2–4^

Since this patient did not present with any signs of infection and no known history of beta-lactam allergic reactions, an adequate level of antibiotic prophylaxis targeting methicillin-sensitive *Staphylococcus aureus* (i.e., common bacteria implicated in cutaneous wound infection), was accomplished with Cephalexin, a first-generation cephalosporin with predominate gram-positive coverage.^24^

After we consoled this patient about the extent of her injury and likely risk for permanent scarring, she expressed optimism concerning the wound care plan we recommended. As this patient’s lesions continued to heal, her need for continuous psychological and physical follow-up and discussion of scar management strategies was still apparent.

During follow-up phone conversations, this woman had confirmed stated that she had the means to go to the wound care center, denying transportation or financial issues as impeding factors. Our further communications with her suggested that she understood her condition, along with the reasoning behind our suggested treatment plan elements. As suggested in this woman’s case, potential factors leading to caregiver-patient miscommunication could contribute to medical non-adherence and be more effectively explored during primary care encounters.^25,26^

## CONCLUSIONS

Partial and full-thickness burn wounds must be carefully assessed and classified in clinic-based and emergent settings, appreciating the likely varied clinical implications in patients with varying BMIs. Along with consideration of immediate fluid resuscitation, needs for more severe burn injuries, etc., clinicians should recognize that partial-thickness (i.e., "second-degree) burn wounds are also likely to cause scarring.

When suffering injuries in cosmetic areas (e g., chest and face), ongoing patient psychological supports (i.e., ongoing affirmation, validation of emotions) may be needed in addition to wound care and scarring management strategies. As in the case of this woman, continued integrated patient care approaches will frequently be indicated.

### Conflicts of Interest

The authors declare no conflicts of interest.
